# Correlation between L‐amino acid transporter 1 expression and 4‐borono‐2‐^18^F‐fluoro‐phenylalanine accumulation in humans

**DOI:** 10.1002/cam4.6635

**Published:** 2023-10-25

**Authors:** Tairo Kashihara, Taisuke Mori, Tetsu Nakaichi, Satoshi Nakamura, Kimiteru Ito, Hiroaki Kurihara, Masahiko Kusumoto, Jun Itami, Seiichi Yoshimoto, Hiroshi Igaki

**Affiliations:** ^1^ Department of Radiation Oncology National Cancer Center Hospital Tokyo Japan; ^2^ Division of Research and Development for Boron Neutron Capture Therapy National Cancer Center Exploratory Oncology Research & Clinical Trial Center Tokyo Japan; ^3^ Department of Pathology and Clinical Laboratories National Cancer Center Hospital Tokyo Japan; ^4^ Department of Medical Physics National Cancer Center Hospital Tokyo Japan; ^5^ Department of Diagnostic Radiology National Cancer Center Hospital Tokyo Japan; ^6^ Department of Diagnostic and Interventional Radiology Kanagawa Cancer Center Yokohama Japan; ^7^ Shin‐Matsudo Accuracy Radiation Therapy Center Shin‐Matsudo Central General Hospital Chiba Japan; ^8^ Department of Head and Neck Surgical Oncology National Cancer Center Hospital Tokyo Japan

**Keywords:** 4‐borono‐2‐^18^F‐fluoro‐phenylalanine positron emission tomography, boron neutron capture therapy, boronophenylalanine, L‐type amino acid transporter 1, neoplasm

## Abstract

**Background:**

The correlation between L‐type amino acid transporter 1 (LAT1) expression and 4‐borono‐2‐^18^F‐fluoro‐phenylalanine (^18^F‐FBPA) accumulation in humans remains unclear. This study aimed to investigate the correlation between LAT1 expression in tumor tissues and ^18^F‐FBPA accumulation in patients with head and neck cancer who participated in a clinical trial of ^18^F‐FBPA positron emission tomography (PET).

**Methods:**

Altogether, 28 patients with head and neck cancer who participated in a clinical trial of ^18^F‐FBPA PET at our institution between March 2012 and January 2018 were included. Correlations between standardized uptake values (SUVs); the maximum SUV (SUV_max_), the mean SUV within a 1 cm^3^ sphere centered at a single point, that is, the SUV_max_ (SUV_peak_), the minimum SUV (SUV_min_), and the intensity of LAT1 expression (maximum and minimum LAT1 expressions) were investigated.

**Results:**

Weak correlations were identified between SUV_max_ and LAT1 maximum score, SUV_min_ and LAT1 maximum score, and SUV_min_ and LAT1 minimum score (*ρ* = 0.427, 0.362, and 0.330, respectively). SUV_max_ and LAT1 minimum score, SUV_peak_ and LAT1 maximum score, and SUV_peak_ and LAT1 minimum score demonstrated moderate correlations (*ρ* = 0.535, 0.556, and 0.661, respectively). Boron neutron capture therapy (BNCT) was performed in 2 of the 4 patients with discrepancies between ^18^F‐FBPA accumulation and intensity of LAT1 expression, and the intensity of LAT1 expression was a better predictor of treatment response.

**Conclusion:**

^18^F‐FBPA accumulation and the intensity of LAT1 expression demonstrated a moderate correlation; however, LAT1 expression may be a better predictor of treatment response of BNCT in patients with discrepancies.

## Graphical abstract

The intensity of L‐type amino acid transporter 1 (LAT1) expression and 4‐borono‐2‐18F‐fluoro‐phenylalanine (18F‐FBPA) accumulation demonstrated a moderate correlation. However, LAT1 expression may be a better predictor of treatment response of boron neutron capture therapy in patients with discrepancies.

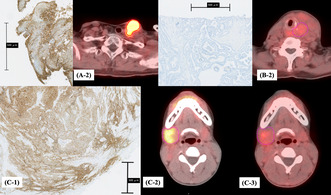

## 1 INTRODUCTION

Boron neutron capture therapy (BNCT) is a radiation therapy using the nuclear reaction of ^10^B(n,α)^7^Li, which occurs when a drug containing ^10^B is concentrated on tumor cells and irradiated with neutrons.^1^, ^2^, ^3^, ^4^, ^5^ α‐Rays and ^7^Li recoil nuclei are high‐linear energy transfer radiation with high biological effects, and their range is almost the same as the cell diameter; thus, cancer cells can be selectively destroyed. The results of a phase II clinical trial of BNCT for unresectable recurrent and locally advanced head and neck cancers^6^, ^7^ revealed a high tumor response rate of 71% with few severe side effects. Consequently, BNCT was approved in Japan in 2020 for the treatment of patients with unresectable recurrent or locally advanced head and neck cancers. Borofalan, formerly known as 4‐borono‐L‐phenylalanine (BPA), was used as ^10^B in the BNCT. Sufficient ^10^B uptake by cancer cells is critical for effective BNCT. The uptake of BPA into cancer cells is mediated by L‐type amino acid transporter 1 (LAT1).^8^, ^9^ The indications for BNCT are generally based on the tumor‐to‐normal tissue ratio and standardized uptake value (SUV) of the tumor using positron emission tomography (PET) with 4‐borono‐2‐^18^F‐fluoro‐phenylalanine (^18^F‐FBPA).^10^, ^11^, ^12^, ^13^, ^14^ In previous studies on glioma cells, a high selectivity of ^18^F‐FBPA for LAT1 expression has been reported.^8^, ^15^ However, ^18^F‐FBPA PET can only predict BPA accumulation at the macroscopic level and cannot assess the heterogeneous cellular‐level uptake of ^18^F‐FBPA in tumor tissues.^16^, ^17^, ^18^ The accumulation of ^18^F‐FBPA in tumor tissues has been reported to be affected by stromal cell density.^19^ However, by examining LAT1 expression in tumor tissues from patients, we can analyze BPA accumulation at the microscopic level and estimate the therapeutic effect of BNCT more accurately.^20^

In this study, the LAT1 expression in tumor tissues of patients with head and neck cancer who underwent ^18^F–FBPA PET/computed tomography (CT) in clinical trials at our institution from 2012 to 2018 was investigated. The first aim of this study was to determine the correlation between ^18^F‐FBPA accumulation in ^18^F‐FBPA PET images and LAT1 expression. The second aim was to characterize the cases with discrepancies in LAT1 expression intensity and ^18^F‐FBPA accumulation.

## 2 MATERIALS AND METHODS

### 2.1 Patient population

**FIGURE 1:**
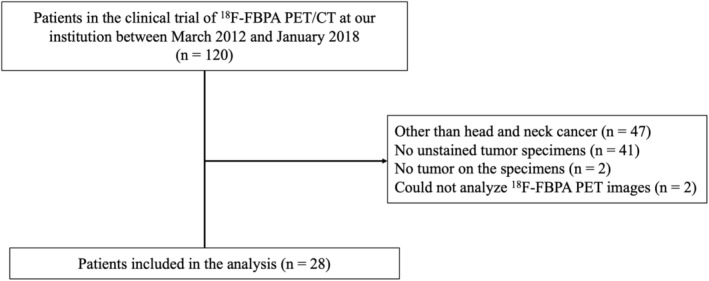
Flowchart of patient inclusion.

A flowchart of the patient inclusion process is presented in Figure 1. Data of 120 patients in the clinical trial of ^18^F‐FBPA PET/CT at our institution between March 2012 and January 2018 were retrieved. Among these patients, those without tumor tissue or those for whom ^18^F‐FBPA PET images could not be analyzed because of a lack of information at the time of imaging, such as the method of ^18^F‐FBPA administration, were excluded. Finally, the data from 28 patients were included in the analysis. The following factors were investigated: age, sex, primary tumor sites, pathology, tissue sampling method (biopsy or surgery), gross tumor volume (GTVs), and the interval between the biopsy date and ^18^F‐FBPA PET/CT imaging date.

### 2.2 Method for evaluating 18F‐FBPA accumulation on 18F‐FBPA PET/CT images

A previous study described the details of the methods of performing an ^18^F‐FBPA PET/CT scan.^21^ ^18^F‐FBPA images were acquired 60 min after injection using a PET/CT scanner (Discovery 600; GE Healthcare, Milwaukee). All ^18^F‐FBPA images were confirmed by a board‐certified radiologist or nuclear physician. SUVs were measured using the AW Volume Share 4.5 software (GE Healthcare, Milwaukee). The GTVs and regions of interest (ROIs) were drawn by a radiation oncologist and a board‐certified radiologist/nuclear medicine technician, respectively. The maximum SUV (SUV_max_) of the tumors in the ROIs was defined as the area with the highest activity. SUV_max_, the mean SUV within a 1 cm^3^ sphere centered at a single point, that is, SUV_max_ (SUV_peak_) and the minimum SUV (SUV_min_) were used to evaluate the intensity of ^18^F‐FBPA accumulation, and kurtosis was used to evaluate heterogeneity.^22^

### 2.3 Method for evaluating LAT1 expression

**FIGURE 2:**
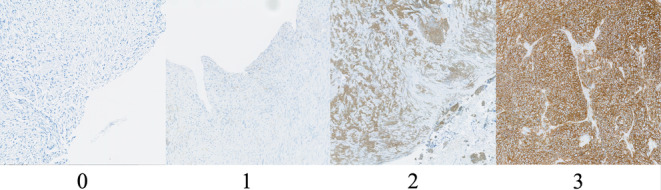
Examples of the L‐type amino acid transporter 1 scoring.

Tumor tissues removed by clinical biopsy or surgery were prepared as 4‐mm‐thick formalin‐fixed, paraffin‐embedded tissue sections. The monoclonal antibody was anti‐LAT1 antibody (4A2, 2 mg/mL) diluted 8000‐fold, and the antigen was removed by microwave treatment at 98°C for 40 min in citrate buffer (pH 6.0, 0.01 mol/L). Immunostaining was performed using a Dako Autostainer Link 48 (Agilent Technologies). The intensity of LAT1 expression was evaluated by a pathologist using a previously reported 4‐point scoring from 0 to 3, with 0 being the weakest expression and 3 being the strongest.^23^ Examples of scoring are demonstrated in Figure 2. The intensity of LAT1 expression was assessed using the maximum and minimum plasma membrane scores of each sample. Heterogeneity was considered present if the maximum and minimum scores differed.

### 2.4 Methods for evaluating the correlations between 18F‐FBPA accumulations and LAT1 expressions (statistical analyses)

Data on LAT1 expression and ^18^F‐FBPA accumulation were collected independently, and the correlation analysis was performed by one physician. Correlations between parameters related to ^18^F‐FBPA accumulation, such as SUV_max_ and SUV_peak_ and SUV_min_, and those related to the intensity of LAT1 expression based on the maximum and minimum scores were investigated. The correlations between the heterogeneity of ^18^F‐FBPA accumulation and LAT1 expression were also investigated. The strength of the correlations was evaluated using the Spearman's rank correlation coefficient. A correlation coefficient (*ρ*) of ≥0.3 was considered correlative. Furthermore, correlations of 0.3 ≤ *ρ* ≤ 0.5, 0.5 ≤ *ρ* ≤ 0.7, and 0.7 ≤ *ρ* were considered weak, moderate, and strong, respectively. To characterize patients with discrepancies between the intensity of LAT1 expression and ^18^F‐FBPA accumulation, detailed data including dynamic ^18^F‐FBPA PET findings were investigated regarding the patients with a maximum LAT1 score of 3 but SUV_peak_ < 2.5 and those with a maximum LAT1 score of 0 but SUV_peak_ ≥ 2.5. Statistical significance was set at *p* < 0.05. All the statistical analyses were performed using the IBM SPSS version 26 software (IBM Corp.).

### 2.5 Ethical approval

All the analyses performed in this study were approved by the appropriate institutional review board (approval number: 2020‐277) and were performed in accordance with the ethical standards of the committee and the 1964 Helsinki Declaration and its later amendments.

## 3 RESULTS

Patient and treatment characteristics are summarized in Table 1. The median age was 56.5 years (range, 6–76 years). Oral cancer was the most common primary cancer. The pathological diagnosis was squamous cell carcinoma in 10 patients (35.7%) and rhabdomyosarcoma in 6 patients (21.4%). The tissue sampling method was biopsy in 12 patients (42.9%) and surgery in 16 patients (57.1%). The median volume of GTVs was 34.4 cm^3^ (1.5–678.1). The LAT1 maximum scores were 0, 1, 2, and 3, and the minimum scores were 7, 5, 6, and 10 for 3, 5, 2, and 18 patients, respectively. If the sphere centered on SUV_max_ extends beyond the GTV, the SUV_peak_ cannot be calculated because it includes nearby normal tissue. Therefore, the SUV_peak_ could not be calculated for six patients. The median SUV_max_, SUV_peak_, and SUV_min_ were 3.70 (range, 1.25–12.42), 3.00 (range, 1.05–10.84), and 0.53 (0.04–1.90). The median kurtosis was −0.23 (−1.06–1.51). Thirteen patients (46.4%) were classified as having a heterogeneous LAT1 expression. Treatment prior to biopsy was chemotherapy plus radiation therapy in 7 patients, chemotherapy in 2 patients, radiation therapy in 2 patients, and surgery in 1 patient; meanwhile, 16 patients were untreated. The median number of days between specimen sampling and ^18^F‐FBPA PET/CT imaging was 166.5 days (range, 4–2410 days). Between specimen sampling and ^18^F‐FBPA PET/CT imaging, 18 patients received treatment: 9 received chemotherapy, 8 received chemotherapy plus radiation therapy, and 1 received radiation therapy.

**TABLE 1:** Patient characteristics.

Characteristic	*N*
Age, median (range)	56.5 (6–76)
Sex	
Men	18
Women	10
Primary tumor site	
Oral cancer	8
Sinonasal cancer	5
Oropharyngeal/hypopharyngeal cancer	5
Orbital tumor	3
Salivary gland cancer	3
Nasopharyngeal cancer	2
Others	2
Pathology	
Squamous cell carcinoma	10
Rhabdomyosarcoma	6
Adenocarcinoma	3
Adenoid cystic carcinoma	3
Melanoma	2
Others	4
Tissue sampling method	
Biopsy	12
Surgery	16
LAT1 maximum score	
0	3
1	5
2	2
3	18
LAT1 minimum score	
0	7
1	5
2	6
3	10
SUV_max_, median (range)	3.70 (1.25–12.42)
SUV_peak_, median (range)	3.00 (1.05–10.84)
SUV_min_, median (range)	0.53 (0.04–1.90)

Abbreviations: LAT1, L‐type amino acid transporter 1; SUV, standardized uptake value; SUV_max_, maximum SUV; SUV_min_, minimum SUV; SUV_peak_, mean SUV within a 1 cm^3^ sphere centered at a single point that is the SUV_max_.

**FIGURE 3:**
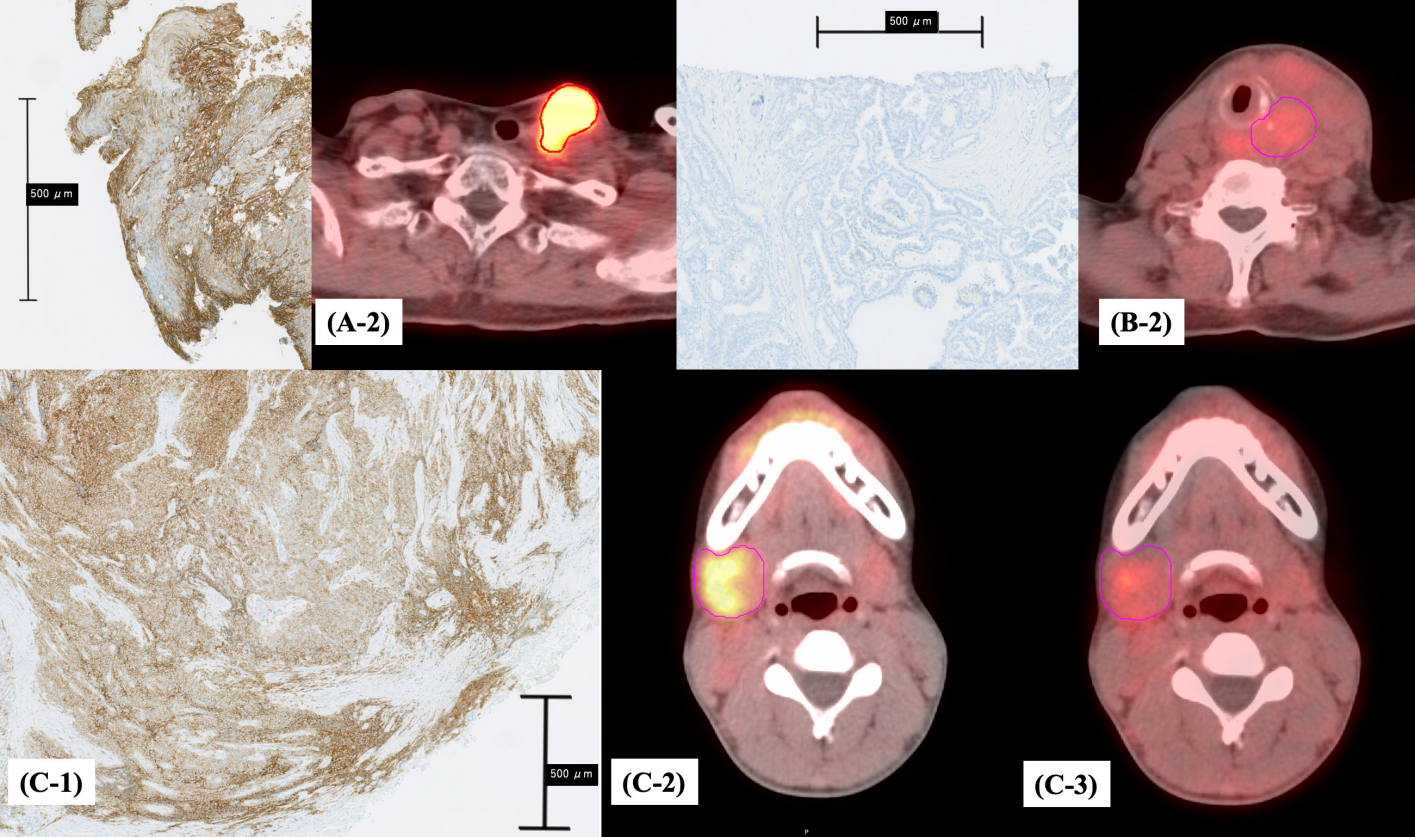
Immunostained images with L‐type amino acid transporter 1 (LAT1) antibody and 4‐borono‐2‐^18^F‐fluoro‐phenylalanine (^18^F‐FBPA) positron emission tomography (PET)/computed tomography (CT) images of the same patients. (A‐1) Maximum and minimum LAT1 expressions were 3. (A‐2) The maximum standardized uptake value (SUV_max_) was high (6.78). (B‐1) Maximum and minimum LAT1 expressions were 0. (B‐2) SUV_max_ was low (2.63). (C) This patient had a discrepancy between the intensity of LAT1 expression and SUV accumulation (Patient A in Table 3). (C‐1) Maximum and minimum LAT1 expressions were 3 and 2, respectively. (C‐2, 3) SUV_max_ at 10 min after ^18^F‐FBPA administration was high (5.01, C‐2), but washed out early and remained low at 1 h (2.65, C‐3).

**FIGURE 4:**
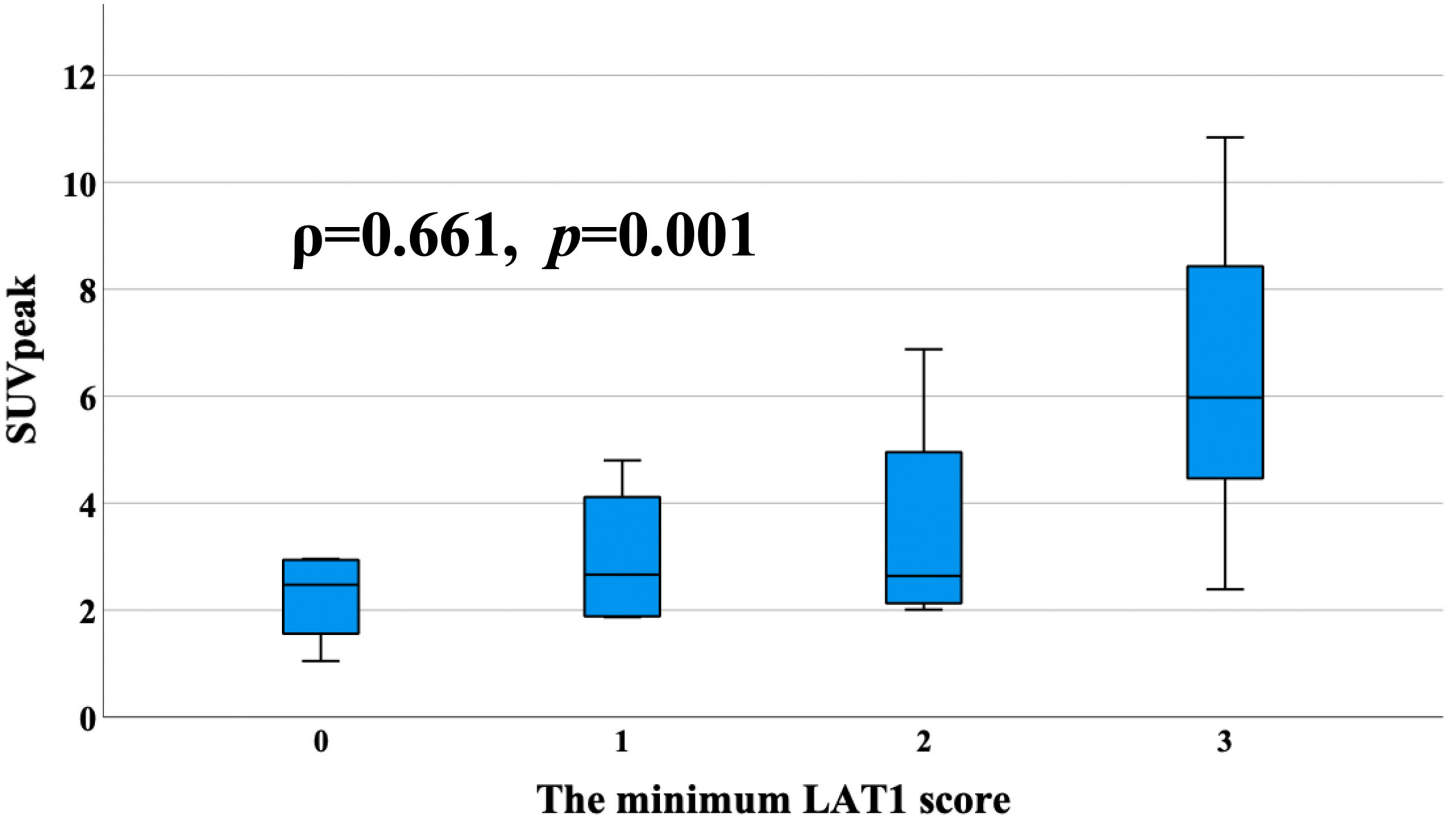
Correlation between the mean standardized uptake value (SUV) within a 1 cm^3^ sphere centered at a single point that is the maximum SUV (SUV_peak_) and the maximum L‐type amino acid transporter 1 (LAT1) score.

The correlations between SUVs and LAT1 scores are presented in Table 2. Immunostained images with LAT1 antibody and ^18^F‐FBPA PET/CT images of the same patients are shown in Figure 3. Weak correlations were identified between SUV_max_ and LAT1 maximum score, between SUV_min_ and LAT1 maximum score, and between SUV_min_ and LAT1 minimum score (*ρ* = 0.427, 0.362, and 0.330, respectively). SUV_max_ and LAT1 minimum score, SUV_peak_ and LAT1 maximum score, and SUV_peak_ and LAT1 minimum score (Figure 4) demonstrated moderate correlations (*ρ* = 0.535, 0.556, and 0.661, respectively). The median kurtosis of SUVs in patients with heterogeneity in LAT1 expression was −0.35 (range, −1.06–1.14), and that in patients without heterogeneity was −0.17 (range, −1.03–1.51). No correlation was identified between the heterogeneity of LAT1 expression and the kurtosis of SUV accumulation (*ρ* = 0.168).

**TABLE 2:** The correlations between the intensity of 4‐borono‐2‐^18^F‐fluoro‐phenylalanine accumulation and L‐type amino acid transporter 1 (LAT1) expression.

Parameters	SUV_max_, median (range)	*ρ*	*p* Value	SUV_peak_, median (range)	*ρ*	*p* Value	SUV_min_, median (range)	*ρ*	*p* Value
LAT1 score									
Maximum	0: 2.63 (2.08–3.50)	**0.427**	**0.023**	0: 2.22 (1.56–2.94)	**0.556**	**0.007**	0: 0.27 (0.24–1.07)	0.362	0.075
	1: 3.59 (1.25–5.38)			1: 2.42 (1.05–4.80)			1: 0.27 (0.04–1.87)		
	2: 3.41 (2.67–4.15)			2: 2.67 (1.90–3.43)			2: 0.52 (0.51–0.53)		
	3: 4.55 (2.06–12.42)			3: 5.67 (2.01–10.84)			3: 0.68 (0.11–1.90)		
Minimum	0: 3.32 (1.25–4.32)	**0.535**	**0.003**	0: 2.48 (1.05–2.96)	**0.661**	**0.001**	0: 0.38 (0.24–1.87)	0.330	0.108
	1: 2.67 (2.05–5.38)			1: 2.67 (1.87–4.80)			1: 0.32 (0.04–0.53)		
	2: 3.82 (2.65–8.19)			2: 2.64 (2.01–6.88)			2: 0.61 (0.30–1.46)		
	3: 6.92 (2.06–12.42)			3: 5.98 (2.39–10.84)			3: 0.85 (0.11–1.90)		

Bolded areas represent factors with *p* < 0.05. Abbreviations: LAT1, L‐type amino acid transporter 1; SUV, standardized uptake value; SUV_max_, maximum SUV; SUV_min_, minimum SUV; SUV_peak_, mean SUV within a 1 cm^3^ sphere centered at a single point that is the SUV_max_.

Patients with discrepancies between ^18^F‐FBPA accumulation and LAT1 expression intensity are summarized in Table 3. Three patients with a LAT1 score of 3 had an SUV_peak_ < 2.5 (Table 3, patients A–C). The pathological diagnoses were adenoid cystic carcinoma, squamous cell carcinoma, and Ewing's sarcoma in one patient each. Two patients demonstrated heterogeneity in LAT1 expression levels. The median number of days between specimen sampling and ^18^F‐FBPA PET/CT imaging was 570 days (range 5–1423 days). The median volume of GTVs was 29.0 cm^3^ (11.3–88.4). In addition, on dynamic ^18^F‐FBPA PET/CT, SUV_max_ at 10 min after ^18^F‐FBPA administration was 5.22, 5.01, and 4.34 for patients A–C, respectively, which was washed out early and remained low at 1 h (Figure 3C). Patient C was treated with BNCT for a primary tumor in the parotid gland after laminectomy of the 12th thoracic metastasis and prior to ^18^F‐FBPA PET imaging; the SUV_max_ was 3.1 for the primary tumor site and 3.0 for the 12th thoracic metastasis site. After BNCT, the tumor significantly shrank but remained in the deep part of the tumor, where neutrons could not reach. By contrast, one patient with a LAT1 score of 0 had an SUV_peak_ ≥ 2.5 (Table 3, patient D). The pathological diagnosis was rhabdomyosarcoma. The number of days between specimen sampling and ^18^F‐FBPA PET/CT imaging was 821 days. The GTV was 207.9 cm^3^. In the patient, no LAT1 expression was observed on the plasma membrane, but staining was detected in the cytoplasm. The patient also underwent an ^18^F‐FBPA PET/CT at the time of postoperative recurrence. The patient was treated with BNCT after ^18^F‐FBPA PET/CT; however, the effect was poor as the tumor grew.

**TABLE 3:** Patients with a discrepancy between the intensity of 4‐borono‐2‐^18^F‐fluoro‐phenylalanine accumulation and L‐type amino acid transporter 1 (LAT1) expression.

Patients	LAT1_max_	LAT1_min_	SUV_max_	SUV_peak_	SUV_min_	GTVs (cm^3^)	Pathology	Days between sampling and PET
A	3	2	2.65	2.01	0.48	11.3	Ewing sarcoma	5
B	3	2	2.89	2.25	0.73	29.0	SCC	1423
C	3	3	3.08	2.39	0.30	88.4	ACC	570
D	0	0	3.50	2.94	0.24	207.9	Rhabdomyosarcoma	821

Abbreviations: ACC, adenoid cystic carcinoma; GTV, gross tumor volume; LAT1, L‐type amino acid transporter 1; LAT1_max_, maximum LAT1; LAT1_min_, minimum LAT1; PET, positron emission tomography; SCC, squamous cell carcinoma; SUV, standardized uptake value; SUV_max_, maximum SUV; SUV_min_, minimum SUV; SUV_peak_, mean SUV within a 1 cm^3^ sphere centered at a single point that is the SUV_max_.

## 4 DISCUSSION

In our study, we demonstrated a correlation between LAT1 expression intensity and SUV accumulation on ^18^F‐FBPA PET/CT images. The strongest correlation was between the LAT1 minimum score and SUV_peak_. The minimum score demonstrating a stronger correlation than the maximum LAT1 score may possibly be because the LAT1 score was evaluated at the microscopic level, and even if the maximum score was high, a large area with a low minimum score may not lead to ^18^F‐FBPA accumulation at the macroscopic level. Therefore, scoring that considers not only the intensity of expression, but also the range, such as the tumor proportion score for programmed cell death ligand 1, may be more useful in predicting the therapeutic effect of BNCT.

In this study, patients with discrepancies in LAT1 expression and ^18^F‐FBPA accumulation were further investigated. All patients with a strong LAT1 expression demonstrated a high accumulation of ^18^F‐FBPA at 10 min, according to further detailed analysis of dynamic ^18^F‐FBPA PET, although ^18^F‐FBPA was washed out early. As 1‐h values are commonly used for ^18^F‐FBPA PET measurements,^12^, ^14^, ^24^ the SUV values were considered low. In a previous study on dynamic ^18^F‐FBPA PET, ^18^F‐FBPA uptake was reported to be washed out early in squamous cell carcinoma, 30 min after ^18^F‐FBPA administration.^21^ Because BPA is administered continuously by intravenous infusion during BNCT, even patients whose ^18^F‐FBPA is washed out early will benefit from BNCT. Patient C had a high LAT1 expression in the surgical specimen of the 12th thoracic metastasis; however, ^18^F‐FBPA accumulation was low, with an SUV_max_ of 3.0. The therapeutic effect of BNCT on primary tumors was high. In patient D, although LAT1 expression was absent, ^18^F‐FBPA accumulation was high, with an SUV_max_ of 3.5. The patient was subsequently treated with BNCT, which did not result in tumor shrinkage. In these cases, LAT1 expression may be more useful in predicting the treatment response after BNCT than ^18^F‐FBPA PET. The small tumor volume and heterogeneity of LAT1 expression in patient A may have influenced the low ^18^F‐FBPA accumulation. Furthermore, a time gap was observed between specimen sampling and ^18^F‐FBPA PET/CT imaging, which may have changed the nature of the tumor.

^18^F‐FBPA PET has been used to predict the therapeutic effects of BNCT using borofalan. In a previous study of ^18^F‐FBPA PET in rat xenograft models of C6 glioma, high selectivity of ^18^F‐FBPA for LAT1 was reported.^15^ Previous studies have identified a correlation between ^18^F‐FBPA accumulation on ^18^F‐FBPA PET by bolus injection and BPA uptake by continuous infusion in mice.^25^, ^26^ Furthermore, Imahori et al.^27^ reported a correlation between ^18^F‐FBPA accumulation on ^18^F‐FBPA PET and ^10^B accumulation in surgical specimens of patients with high‐grade gliomas. However, Aihara et al.^28^ reported that the SUV_max_/SUV of normal tissue (SUV_N_), mean SUV, SUV_min_, and Vo2.5 (tumor volume ratio of SUV_max_/SUV_N_ ≥ 2.5) were not prognostic factors for complete response rate. In their study, SUV_min_/SUV_N_ was the only significant factor; however, SUV_min_ and SUV_N_ are difficult to use as indicators because they are less reproducible owing to their small range of values and their dependence on how the ROI is set.^22^ By contrast, a previous study reported that increased LAT1 expression also increased the uptake capacity of BPA by tumors.^29^ Onishi et al.^30^ reported that the overexpression of LAT1 in cancer cells enhanced the therapeutic effect of BNCT in vitro. However, to the best of our knowledge, this is the first report of a correlation between LAT1 expression in human tumor specimens and ^18^F‐FBPA accumulation in human tumors undergoing ^18^F‐FBPA PET.

To assess the biological effects of BNCT accurately, it is essential to consider the intercellular heterogeneity of ^10^B distributions.^31^ In the present study, no correlation was identified between ^18^F‐FBPA PET and LAT1 expression and heterogeneity. The following are possible reasons for this: First, as ^18^F‐FBPA PET is a macroscopic assessment, heterogeneity may not be assessed at the cellular level. Second, the biopsy specimens were assessed for only a part of the tumor, and heterogeneity across the entire tumor may not have been assessed. Both macroscopic and microscopic evaluation by LAT1 expression and ^18^F‐FBPA PET may solve these problems. Third, the different timings of biopsy and ^18^F‐FBPA PET/CT imaging may have changed the characteristics of heterogeneity.

This study has two limitations. First, a time lag occurred between specimen collection and ^18^F‐FBPA PET/CT imaging in some cases. Second, it was a retrospective study with a small sample size, which may include some bias. Prospective studies investigating the correlation between LAT1 expression and treatment response in patients undergoing BNCT are required.

In conclusion, the evaluation of LAT1 expression by specimen sampling correlated with ^18^F‐FBPA accumulation on ^18^F‐FBPA PET/CT imaging; however, in some patients with discrepancies between them, LAT1 expression may be more useful in predicting BNCT treatment response. The LAT1 expression score is a good candidate biomarker for predicting BNCT treatment response and awaits validation by studies investigating its correlation with treatment response in patients undergoing BNCT.

## AUTHOR CONTRIBUTIONS

**Tairo Kashihara:** Conceptualization (lead); formal analysis (lead); funding acquisition (lead); investigation (lead); methodology (lead); project administration (lead); resources (lead); supervision (lead); visualization (lead); writing – original draft (lead); writing – review and editing (lead). **Taisuke Mori:** Investigation (equal); methodology (equal); resources (equal); visualization (equal); writing – review and editing (supporting). **Tetsu Nakaichi:** Investigation (equal); methodology (equal); writing – review and editing (supporting). **Satoshi Nakamura:** Conceptualization (equal); methodology (equal); project administration (supporting); validation (supporting); writing – review and editing (supporting). **Kimiteru Ito:** Methodology (supporting); resources (supporting); writing – review and editing (supporting). **Hiroaki Kurihara:** Methodology (supporting); resources (supporting). **Masahiko Kusumoto:** Project administration (supporting). **Jun Itami:** Conceptualization (equal); methodology (equal); resources (equal); writing – review and editing (supporting). **Seiichi Yoshimoto:** Resources (equal). **Hiroshi Igaki:** Conceptualization (equal); methodology (equal); project administration (equal); supervision (equal); writing – review and editing (supporting).

## FUNDING INFORMATION

This work was supported in part by the Grant from Japanese Society for Radiation Oncology (JASTRO) and Takeda Science Foundation.

## CONFLICT OF INTEREST STATEMENT

Mr. Nakamura reports research funds from Cancer Intelligence Care Systems, Inc. outside the submitted work. Dr. Kusumoto reports honoraria from AstraZeneca K.K., Daiichi‐Sankyo Inc., and Daiichi‐Sankyo Co., Ltd., and research funds from Cannon medical systems outside the submitted work. Dr. Itami reports advisor remuneration from HekaBio and SunnyHealth and research funds from Palette Science. Dr. Yoshimoto reports research funds from CHUGAI PHARMACEUTICAL Co., Ltd. outside the submitted work. Dr. Igaki reports research grant from Elekta K.K., Itochu, and AstraZeneca outside the submitted work. The other authors declare that they have no known competing financial interests or personal relationships that could have appeared to influence the work reported in this paper.

## ETHICS STATEMENT

Approval of the research protocol by an Institutional Reviewer Board: All analyses performed in this study were approved by the Committee's review board at our institution (approval number, 2020‐277) and were in accordance with the ethical standards of the committee and the 1964 Helsinki Declaration and its later amendments. Informed consent: Informed consent was obtained in the form of opt‐out on the website. Those who rejected were excluded. Registry and the Registration No. of the study/trial: N/A. Animal Studies: N/A.

## Data Availability

The data that support the findings of this study are available from the corresponding author upon reasonable request.
